# From Sea to Science: Coral Aquaculture for Sustainable Anticancer Drug Development

**DOI:** 10.3390/md22070323

**Published:** 2024-07-19

**Authors:** Hung-Yu Lin, Tsen-Ni Tsai, Kai-Cheng Hsu, Yu-Ming Hsu, Lin-Chien Chiang, Mohamed El-Shazly, Ken-Ming Chang, Yu-Hsuan Lin, Shang-Yi Tu, Tony Eight Lin, Ying-Chi Du, Yi-Chang Liu, Mei-Chin Lu

**Affiliations:** 1 School of Medicine, College of Medicine, I-Shou University, Kaohsiung 824, Taiwan; 2 Division of Urology, Department of Surgery, E-Da Cancer Hospital, I-Shou University, Kaohsiung 824, Taiwan; 3 Division of Hematology-Oncology, Department of Internal Medicine, Kaohsiung Medical University Hospital, Kaohsiung 807, Taiwan; 4 Graduate Institute of Marine Biology, National Dong Hwa University, Pingtung 944, Taiwan; 5 Graduate Institute of Cancer Biology and Drug Discovery, College of Medical Science and Technology, Taipei Medical University, Taipei 110, Taiwan; 6 Ph.D. Program for Cancer Molecular Biology and Drug Discovery, College of Medical Science and Technology, Taipei Medical University, Taipei 110, Taiwan; 7 Research Center for Precision Environmental Medicine, Kaohsiung Medical University, Kaohsiung 807, Taiwan; 8 Department of Pharmacognosy, Faculty of Pharmacy, Ain-Shams University, Organization of African Unity Street, Abassia, Cairo 11566, Egypt; 9 Department of Pharmacy and Master Program, Tajen University, Pingtung 907, Taiwan; 10 National Museum of Marine Biology and Aquarium, Pingtung 944, Taiwan

**Keywords:** apoptosis, epithelial–mesenchymal transition (EMT), prostate cancer, tubulin polymerization, 13-acetoxysarcocrassolide

## Abstract

Marine natural products offer immense potential for drug development, but the limited supply of marine organisms poses a significant challenge. Establishing aquaculture presents a sustainable solution for this challenge by facilitating the mass production of active ingredients while reducing our reliance on wild populations and harm to local environments. To fully utilize aquaculture as a source of biologically active products, a cell-free system was established to target molecular components with protein-modulating activity, including topoisomerase II, HDAC, and tubulin polymerization, using extracts from aquaculture corals. Subsequent in vitro studies were performed, including MTT assays, flow cytometry, confocal microscopy, and Western blotting, along with in vivo xenograft models, to verify the efficacy of the active extracts and further elucidate their cytotoxic mechanisms. Regulatory proteins were clarified using NGS and gene modification techniques. Molecular docking and SwissADME assays were performed to evaluate the drug-likeness and pharmacokinetic and medicinal chemistry-related properties of the small molecules. The extract from *Lobophytum crassum* (LCE) demonstrated potent broad-spectrum activity, exhibiting significant inhibition of tubulin polymerization, and showed low IC_50_ values against prostate cancer cells. Flow cytometry and Western blotting assays revealed that LCE induced apoptosis, as evidenced by the increased expression of apoptotic protein-cleaved caspase-3 and the populations of early and late apoptotic cells. In the xenograft tumor experiments, LCE significantly suppressed tumor growth and reduced the tumor volume (PC3: 43.9%; Du145: 49.2%) and weight (PC3: 48.8%; Du145: 7.8%). Additionally, LCE inhibited prostate cancer cell migration, and invasion upregulated the epithelial marker E-cadherin and suppressed EMT-related proteins. Furthermore, LCE effectively attenuated TGF-*β*-induced EMT in PC3 and Du145 cells. Bioactivity-guided fractionation and SwissADME validation confirmed that LCE’s main component, 13-acetoxysarcocrassolide (13-AC), holds greater potential for the development of anticancer drugs.

## 1. Introduction

Historically, human medicine has relied heavily on plants and animals, harnessing their secondary metabolites for therapeutic purposes. Secondary metabolites, although not essential for the organism’s survival, carry out significant biological activities and have been utilized as dietary supplements and therapeutic agents. Ancient texts like Shennong’s Classic of Materia Medica and the Compendium of Materia Medica by Li Shizhen document numerous marine organisms and their medicinal properties [1]. Recently, marine natural products have been shown to show potent biological activity, with antitumor properties surpassing those of terrestrial sources, largely due to their unique stereochemical structures and special elemental compositions [1].

Soft corals are a prolific source of terpenoids, especially cembrane diterpenoids, which exhibit a broad spectrum of pharmacological activities, including anti-inflammatory, anticancer, antibacterial, and immunomodulatory effects [2,3]. Taiwan, boasting one of the world’s highest densities and diversities of marine corals, has significant expertise in developing and establishing aquaculture systems [4]. This technical prowess positions Taiwan to be able to advance sustainable marine environment conservation and develop marine-derived medicines targeting many diseases, especially cancer.

Topoisomerases are crucial enzymes in the DNA metabolism, making them prime targets for anticancer drugs. The pursuit of topoisomerase inhibitors has become a significant focus in cancer drug development. Over the past decade, researchers have identified, synthesized, and evaluated a variety of novel bioactive molecules targeting topoisomerases. These inhibitors have been categorized into several structural classes, including N-heterocycles, quinones, flavonoids, diterpenes, coumarins, lignans, polyphenols, fatty acids, and metal complexes [5,6,7,8,9,10,11,12,13].

Despite the prevalence of topoisomerase poisons, such as etoposide, in cancer therapy, their associated cardiotoxicity, resistance issues, and potential for causing secondary malignancies necessitate safer alternatives. Catalytic inhibitors, which block the enzyme’s function without causing DNA damage, represent a promising research direction [14,15,16,17,18]. Our research identified a marine sesquiterpenoid, 13-acetoxysarcocrassolide (13-AC), which competitively inhibits topoisomerase and the N-terminal ATP binding site of heat shock proteins. This compound holds potential as a less toxic anticancer agent, particularly for blood, oral, and prostate cancers [14]

Microtubules, composed of α-tubulin and β-tubulin subunits, are integral to various cellular functions, including cell shape, division, and intracellular transport. The disruption of tubulin polymerization is a key strategy in cancer treatment as it interferes with cell division [19]. Antitubulin drugs, derived from plants, microbes, and marine organisms, either stabilize or destabilize tubulin. Notable stabilizers, such as Taxol and vinca alkaloids, are widely used in clinical settings [20]. Given the critical role of tubulin in cellular processes and tumorigenesis, targeting tubulin polymerization remains a promising approach in anticancer drug development.

Histone deacetylases (HDACs) regulate gene expression by altering the chromatin structure. HDAC inhibitors (HDACis) are a promising class of anticancer agents, especially when designed as multi-target hybrids that inhibit multiple cancer pathways. These hybrid molecules have garnered significant interest due to their potential to reduce side effects and enhance therapeutic efficacy. Recent developments have seen several HDACi hybrids, such as CUDC-101 and CUDC-907, enter clinical trials, underscoring their therapeutic potential [21]. The research into HDAC inhibitors continues to be a vibrant field, with a focus on developing agents that can synergistically improve the effectiveness of other anticancer drugs.

Epithelial–mesenchymal transition (EMT) is a process where epithelial cells acquire mesenchymal traits, enhancing their motility. This transition is crucial during embryonic development, wound healing, and cancer metastasis. EMT enables cancer cells to invade surrounding tissues and disseminate to distant organs [22,23]. More than 90% of cancer-related deaths are caused by metastatic cancer diseases. Given that over 90% of cancer-related deaths are due to metastasis, understanding and targeting EMT mechanisms are critical for developing therapies to inhibit cancer spread [24,25].

This study investigates the anticancer potential of extracts from aquaculture soft corals, focusing on their effects on topoisomerase II, HDAC, and tubulin polymerization. Using in vitro and in vivo assays, we evaluated the extracts’ efficacy against prostate cancer cells and their ability to inhibit cancer cell metastasis and EMT. Specifically, we assessed whether these extracts could inhibit EMT-related protein expression in the presence or absence of TGF-β, an EMT inducer. The goal is to explore the potential of these marine extracts as anticancer and anti-metastatic agents.

## 2. Results

### 2.1. Effects of Different Crude Extracts on the Cell Growth of Leukemia and Prostate Cancer Cells

**Figure 1:**
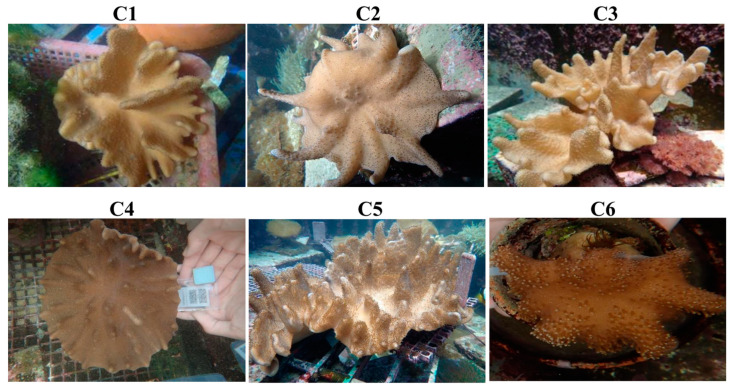
Morphology of aquaculture soft corals.

In this study, more than 180 soft corals in the aquaculture system were extracted using ethyl acetate to screen out their cytotoxicity against leukemic and prostate cancer cells. The results showed that six extracts (Figure 1) inhibited the survival of more than half of the cancer cells at the highest dose (40 μg/mL) for 72 treatments (Table 1). C2 showed the most potent activity, with IC_50_ values of less than 0.16 and 0.33 ± 0.05 μg/mL for leukemia Molt4 and K562 cells, respectively. C5 was the most active extract against prostate Du145 and LNCaP cells, with IC_50_ values of 4.96 ± 0.04 and 2.76 ± 0.34 μg/mL, respectively. The use of any of the six crude extracts at a high dose (40 μg/mL) inhibited almost 90% of growth in four cancer cells, except C3 and C4 for Du145 and LNCaP cells, which resulted in IC_50_ values of over 30 μg/mL.

### 2.2. Effects of the Different Crude Extracts on the Function of Topoisomerase II

**Figure 2:**
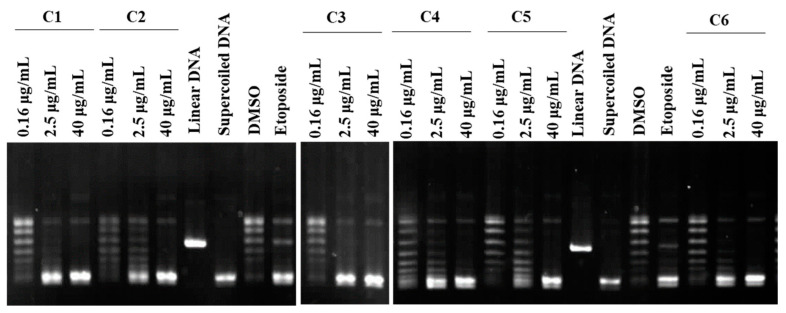
Effects of the six coral extracts (C1–C6) on topoisomerase II activity. Supercoiled DNA was pre-incubated with DNA topoisomerase IIα, and the indicated compounds were added (0.16, 2.5, and 40 μg/mL). After incubation and agarose gel electrophoresis, the bands of linear DNA were analyzed. The result indicated that C1–C6 inhibited TOPO II activity in a dose-dependent manner. Linear and supercoiled DNA were marked. Etoposide was used as the positive control.

The Food and Drug Administration (FDA)-approved chemotherapeutic agents doxorubicin and paclitaxel are topoisomerase inhibitors [26]. A cell-free system assay can be used to detect whether the activity of human topoisomerase II is affected by these coral extracts. The results showed that C1 and C3 increased the level of supercoiled DNA at a dose of 0.16 μg/mL. This indicated that extracts C1 and C3 could diminish the function of topoisomerase II at low doses, and at a dose of 2.5 μg/mL, all the extracts significantly inhibited the formation of relaxed DNA induced by topoisomerase II, while all extracts inhibited topoisomerase II at a high dose of 40 μg/mL (Figure 2). All these extracts dose-dependently decreased the catalytic activity of topoisomerase II, with IC_50_ values of 0.16, 0.55, 0.17, 2.93, 3.10, and 2.35 μg/mL, respectively. Therefore, topoisomerase II could be a potent target of the six coral extracts.

### 2.3. Effects of Different Crude Extracts on HDAC Activity

**Figure 3:**
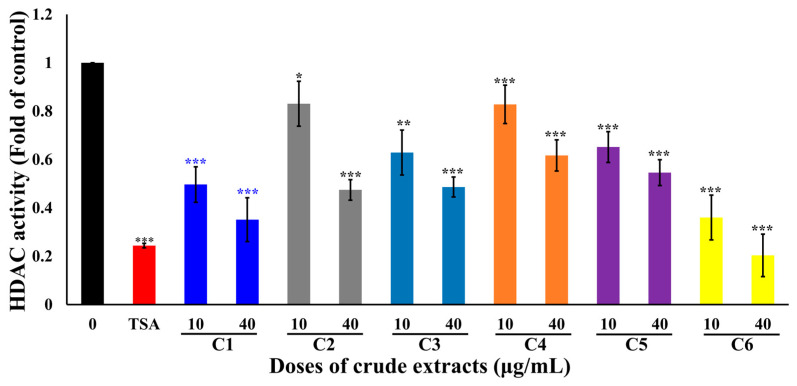
The effect of six coral extracts on histone deacetylase (HDAC) activity in a cell-free system. Trichostatin A (TSA) is a potent reversible inhibitor of HDAC that was used as the positive control. Relative deacetylation of histone was decreased by the treatment with marine organism extracts (10 and 40 µg/mL). (* *p* < 0.05; ** *p* < 0.01; *** *p* < 0.005).

The inhibition of HDAC is a promising target of cancer therapeutics. We evaluated the effect of six coral extracts on HDAC activity. Treatment with six coral extracts at 10 and 40 μg/mL exhibited moderate inhibition of HDAC, with 0.49 ± 0.07 and 0.35 ± 0.09; 0.83 ± 0.09 and 0.47 ± 0.04; 0.88 ± 0.03 and 0.48 ± 0.02; 0.63 ± 0.09 and 0.48 ± 0.04; 0.82 ± 0.08 and 0.62 ± 0.06; and 0.65 ± 0.06 and 0.55 ± 0.05, respectively. The HDAC inhibitor, trichostatin (TSA), significantly inhibited HDAC activity at 300 μM to 0.24 ± 0.01-fold in comparison with the control (Figure 3). The results indicated that the antiproliferative activity of the six coral extracts does not involve HDAC inhibition. The C6 extract showed a comparable activity to trichostatin at 40 μg/mL.

### 2.4. Effects of Different Crude Extracts on the Activity of tubulin Polymerization

**Figure 4:**
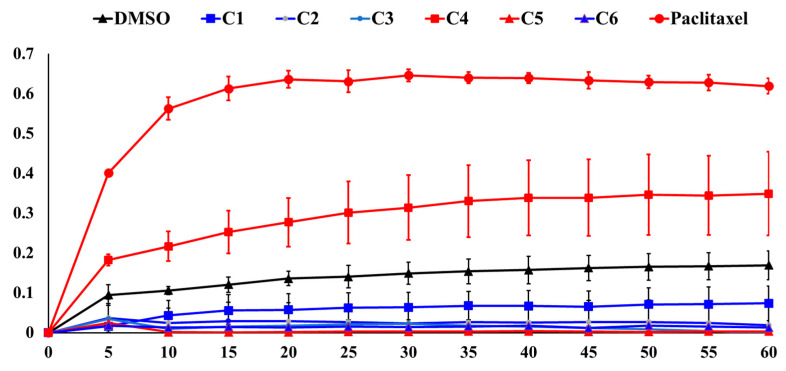
The effects of the coral extracts (4 µg/mL) on the kinetic tubulin polymerization in a cell-free system. Paclitaxel is a tubulin-binding agent and was used as the positive control. The time course of tubulin polymerization and depolymerization at 37 °C was measured in the presence of vehicle (DMSO) or marine organism extracts as indicated. The effect was measured using an ELISA reader at 350 nm. The result indicated that C4 upregulated the level of tubulin polymerization. C1, C2, C3, C5, and C6 upregulated the level of tubulin depolymerization.

Taxol, vinca, and colchicine-binding site analogs are therapeutically active anticancer drugs targeting tubulin polymerization [20]. We determined the effects of the six coral extracts on tubulin polymerization. Tubulin polymerization assays are used to study the effects of drugs and proteins on tubulin assembly and measure the ability of tubulin to polymerize into microtubules by observing changes in the optical density at a specific wavelength (OD 350 nm) [27]. As shown in Figure 4, paclitaxel, a classic tubulin-stabilizing agent, induced time-dependent 4.24-, 5.31-, 5.08-, and 4.67-fold tubulin accumulation compared with the control after 5, 10, 15, and 20 min, respectively. In addition, 1.93-, 2.05-, 2.97-, and 2.03-fold tubulin accumulation was also observed in the presence of C4 extract (4 μg/mL) compared with the control after 5, 10, 15, and 20 min, respectively. Nevertheless, the treatment with C1–C3 and C5–C6 extracts (4 μg/mL) decreased tubulin accumulation 0.17-, 0.39-, 0.37-, 0.22-, and 0.19-fold after 5 min. All treatments reached steady-state equilibrium status after 10 min. Less than 1% of tubulin accumulation was observed with the treatment of C5 extract after 10 min. These results indicated that tubulin could be a potent target of the six coral extracts.

### 2.5. Effect of C5 Extract (LCE) on Cell Growth, Colony Formation, and Apoptosis in Prostate Cancer Cells

**Figure 5:**
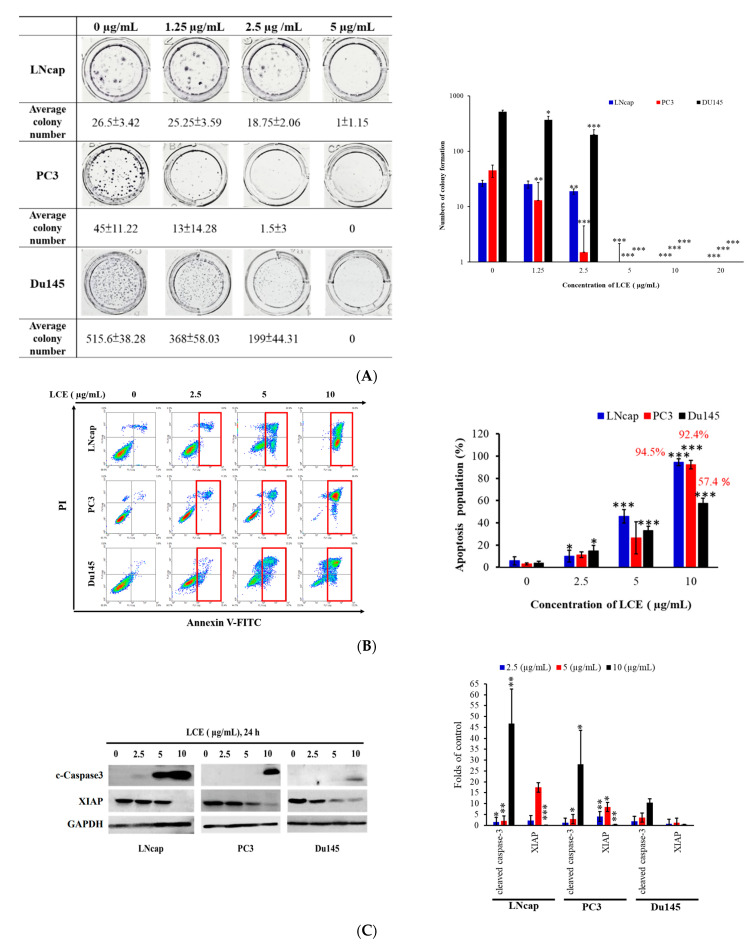
The inhibitory effect of LCE on growth, colony formation, and apoptosis induction in prostate cancer cells. (**A**) Three different cancer cells were treated with different concentrations of LCE in the colony formation assay. (**B**) Apoptosis induction was assessed with annexin-V/PI staining using flow cytometric analysis. (**C**) The expression of apoptosis-regulated proteins was determined with Western blotting analysis. PCa cells were treated with different doses of LCE for 24 h. Glyceraldehyde-3-phosphate dehydrogenase (GAPDH) was used as the loading control. Quantitative results are presented as means ± SD of three independent experiments (* *p* < 0.05; ** *p* < 0.01; *** *p* < 0.005).

Prostate cancer is the fifth leading cause of death in the world, and it is also the second most diagnosed cancer in men, with a high mortality rate. It is usually discovered after being metastasized to other organs, rendering the available treatments ineffective [28]. C5 extract (*Lobophytum crassum* extract, LCE), which showed the most promising antitubulin and antiproliferation activity, was further evaluated against invasive prostate cancer cells using in vitro and in vivo models. The cytotoxic activity of LCE extract was first detected by means of an MTT assay to evaluate its effect on the proliferation of human prostate cancer cells (LNcap, PC3, Du145), and the IC_50_ values were calculated. The results showed that the survival rate of prostate cancer cells decreased when we increased the dose and duration of treatment. The IC_50_ values were 5.70 ± 1.37, 4.14 ± 0.48, and 5.27 ± 2.19 μg/mL after 24 h and 2.76 ± 0.341, 1.92 ± 0.09, and 4.96 ± 0.04 μg/mL at 72 h, respectively (Table 2). Since the MTT results showed that LCE inhibited the growth rate of human prostate cancer cells, the next step was to explore whether different doses of LCE would affect the ability of prostate cancer cells to form colonies under long-term treatment. The results showed that the colony formation of PC3 and Du145, as well as LNCap cells, was significantly decreased at 1.25 μg/mL and 2.5 μg/mL, respectively (Figure 5A). In addition, the apoptosis of LNcap, PC3, and Du145 was dose-dependently induced to 94.5, 92.4, and 57.4% at 10 μg/mL of LCE using flow cytometric analysis (Figure 5B). The results of the Western blotting assay showed that the activation of caspase-3 was increased and the expression of the X-linked inhibitor of apoptosis protein (XIAP) was attenuated at 5 and 10 μg/mL of LCE (Figure 5C). Based on the above results, it can be concluded that LCE could induce apoptosis of prostate cancer cells.

### 2.6. Effect of LCE on Cancer Cellular Invasion Induced with TGFβ

**Figure 6:**
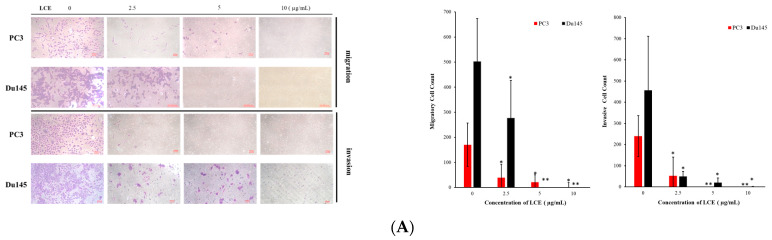

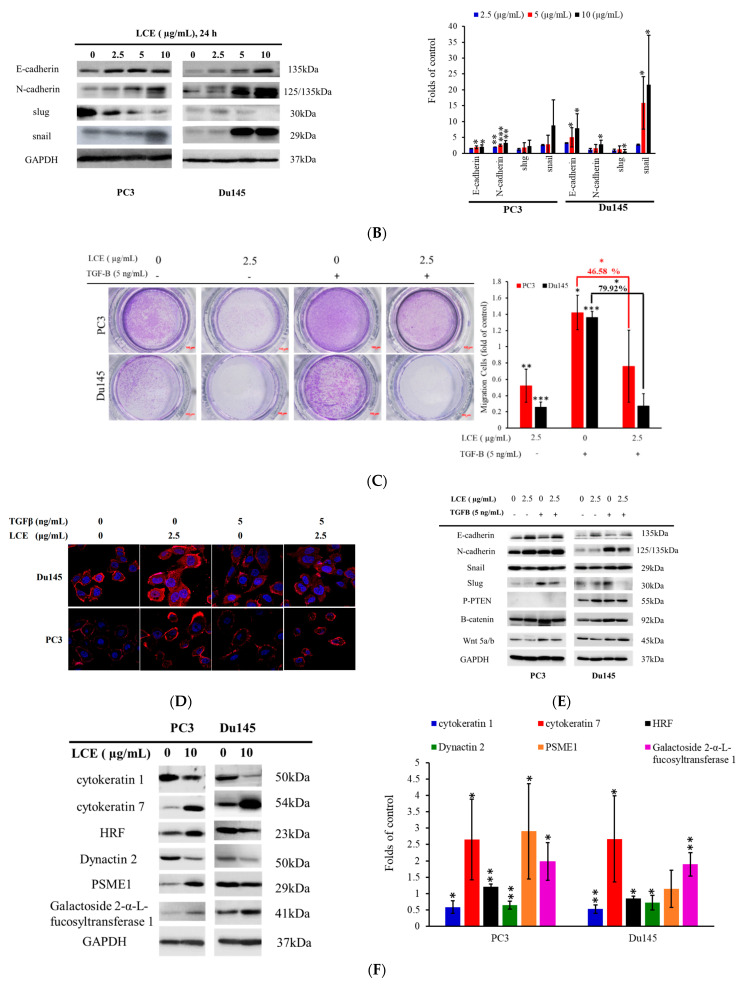
The effect of LCE on the migration and invasion of prostate cancer PC3 and Du145 cells. (**A**) The effect of LCE on the migration and invasion of PC3 and Du145 cells, demonstrated by transwell assay. Quantitative representation of the migration and invasion cells after LCE treatment for 48 and 72 h. (**B**) LCE treatment reduced EMT-related proteins. The expression of the EMT biomarker was determined with a Western blotting assay. PC3 and Du145 cells were treated with different doses of LCE for 24 h. (**C**) The migration of PC3 and Du145 cells was analyzed by means of a transwell assay. Quantitative representation of migration cells after LCE and TGF-β treatment for 48 h. (**D**) The effect of LCE treatment on the expression of E-cadherin, demonstrated using a fluorescent confocal assay. Scale bar: 10 µm. (**E**) The effect of LCE treatment on the expression of EMT-related biomarkers, detected by Western blotting assay. PC3 and Du145 cells were treated with TGF-β (5 ng/mL) and LCE (2.5 μg/mL) for 24 h. (**F**) The effect of LCE treatment on the expression of targeted proteins, detected with a Western blotting assay. PC3 and Du145 cells were treated with LCE (10 μg/mL) for 6 h. GAPDH was used as the loading control. Scale bar, 100 μm. Quantitative results are presented as means ± SD of three independent experiments (* *p* < 0.05; ** *p* < 0.01; *** *p* < 0.005).

A transwell assay is commonly used to mimic the migration and invasion of cells in organisms. It also demonstrates and analyzes the cells’ ability to move in a certain direction in response to various chemoinducers, such as chemokines, growth factors, lipids, or nucleotides [29]. PC3 and Du145 were used to detect the effect of LCE on cell migration and invasion by means of a transwell assay. The migration results showed that the cells’ migration to the lower layer of PC3 and Du145 decreased with the increase in the dose of LCE after 48 h of treatment. The quantification graph showed that the migration activity of both PC3 and Du145 cells was significantly reduced with 2.5 μg/mL of LCE (Figure 6A). The quantification graph of the invasion showed that LCE treatment significantly inhibited the invasion of PC3 and Du145. A Western blotting assay was used to investigate the effect of LCE on the expression of EMT-related proteins in PC3 and Du145 cells. The results showed that the adhesion protein E-cadherin, which maintains epithelial cell properties, was increased in PC3 and Du145 following LCE treatment, especially in Du145, and the transcription factor Slug was reduced (Figure 6B). Despite the presence of the protein N-cadherin and the transcription factor Snail in the mesenchymal cells—the hallmark of EMT—E-cadherin was lost, which is an important marker of epithelial cells [30]. LCE showed a potential inhibitory effect against the metastasis and invasion of prostate cancer cells.

In tumorigenesis and metastasis, the transforming growth factor-β (TGF-β) promotes tumor invasion and metastasis by means of EMT induction [31]. The metastatic invasion activity of the cells is induced by TGF-β. LCE extract was added to detect whether it could influence the transformation of EMT in prostate cancer cells. According to the migration results, LCE treatment (2.5 μg/mL) alone reduced the migration ability in PC3 and Du145 cells, while the number of cell migrations increased by 1.3~1.4 times following TGF-β treatment. The inhibition of cell metastasis was maintained after LCE (2.5 μg/mL) and TGF-β treatment in comparison with the TGF-β group alone. The combined treatment with TGF-β and LCE attenuated metastasis by 46.58% and 79.92% compared with TGF-β treatment, indicating that LCE inhibited metastasis induced by TGF-β treatment. In addition, TGF-β induced an increase in the expression of N-cadherin in prostate cancer cells and decreased the expression of E-cadherin, but it upregulated the performance associated with other EMT biomarkers [32]. LCE treatment reversed the expression of E- and N-cadherin induced by TGF-βin PC3 and Du145 cells, as demonstrated by fluorescent and Western blotting assays, indicating that LCE treatment inhibited the migration and invasion of cancer cells while increasing the expression of E-cadherin and decreasing the expression of N-cadherin (Figure 6D,E).

The proteomic and LC-MS/MS analysis identified six proteins that were changed in LCE-treated PC3 and Du145 cells (Table 3). To verify these proteomic results, a Western blotting assay was used to further determine the expression of six proteins in PC3 and Du145 cells. As shown in Figure 6F, the expression of the six proteins, keratin 1 and 7, dynactin subunit 2 isoform 1 (dynactin 2), proteasome activator compels subunit 1 isoform 1(PSME1), galactoside α-(1,2)-fucosyltransferase 1, and translationally controlled 1 (HRF), was changed in PC3 and Du145 cells after LCE treatment, demonstrating that keratin 1 and 7, dynactin 2, and galactoside α-(1,2)-fucosyltransferase 1 were the principle regulators of the motility and polarity of LCE.

### 2.7. Effect of LCE on Gene Profiling

**Figure 7:**
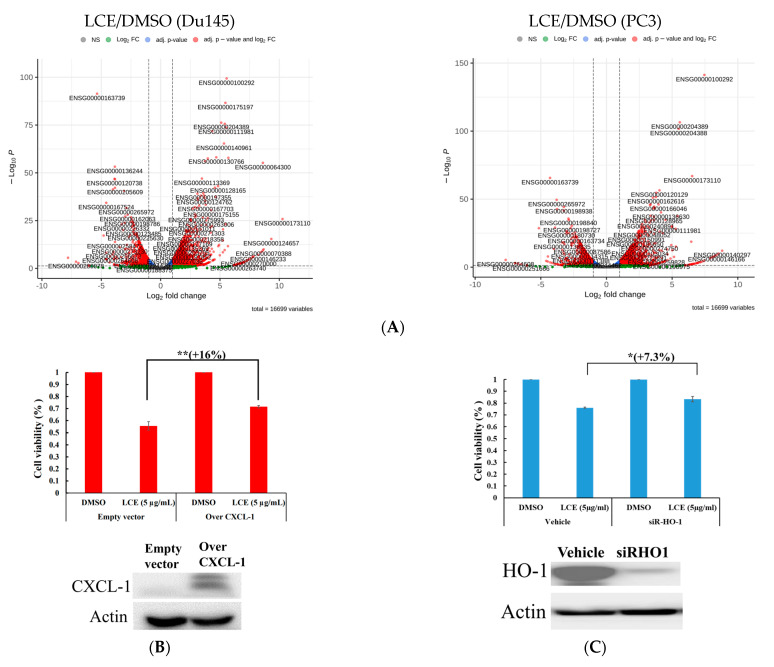
Effect of HO-1 and CXCL1 on the cytotoxic effect of LCE. (**A**) Comparison of genome differences in the two groups of PC3 or Du145 cells treated with LCE using NGS analysis. (**B**,**C**) present the gene modification of CXCL1 (overexpression), and HO-1 (siRNA) was carried out to assess the viability inhibited by LCE treatment. Quantitative results are presented as means ± SD of three independent experiments (* *p* < 0.05; ** *p* < 0.01).

Next-generation sequencing (NGS) has advanced in the scientific arena in the last few years owing to its high throughput, low cost, scalability, and applicability in different biological disciplines. NGS analyses with FASTQ, HISAT2, FeatureCounts, whole-genome, transcriptome, and exome sequencing were collected and compared with the human reference genome [33]. The volcano plot allowed us to check the differences in the performance of genomes among two groups, as well as the statistical significance of these differences. The X coordinate is a multiple of the difference (fold change) (Figure 7A). Of 16,699 variables, the top 10 target proteins are listed in Table 4. In addition, HMOX1 (heme oxygenase 1, HO-1), HSPA1A (HSP70), and HSPA1B (HSP70) simultaneously increased about 5.56- and 7.46-; 5.10- and 5.58-; and 4.34- and 5.50-fold (Log2FoldChange); however, CXCL1 (C-X-C motif chemokine ligand 1) decreased about 5.35- and 4.30-fold compared with the negative control (Log2FoldChange) in PC3 and Du145 cells, respectively, following LCE treatment (Table 4). Based on the above NGS results, HO-1 and CXCL1 were considered potent regulators of the proliferation inhibition of prostate cancer cells induced by LCE treatment. Gene modification was used to verify the intricate mechanisms by which HO-1 or CXCL1 was involved in the cytotoxic effect of LCE. CXCL1 overexpression experiments revealed that the overexpression of CXCL1 led to an augmented increase in viability by 16% compared with the sole LCE treatment in Du145 cells (Figure 7B). Additionally, SiRNA with HO-1 experiments illuminated that the knockdown of HO-1 led to a significant increase in viability by 7.3% compared with the sole LCE treatment in Du145 cells (Figure 7C). These findings suggested that these two proteins possessed the potential to promote the cytotoxic effect of LCE.

### 2.8. 13-Acetoxysarcocrassolide

*Is the Active Component of LCE and a Potential Candidate for Tubulin Inhibition*

**Figure 8:**
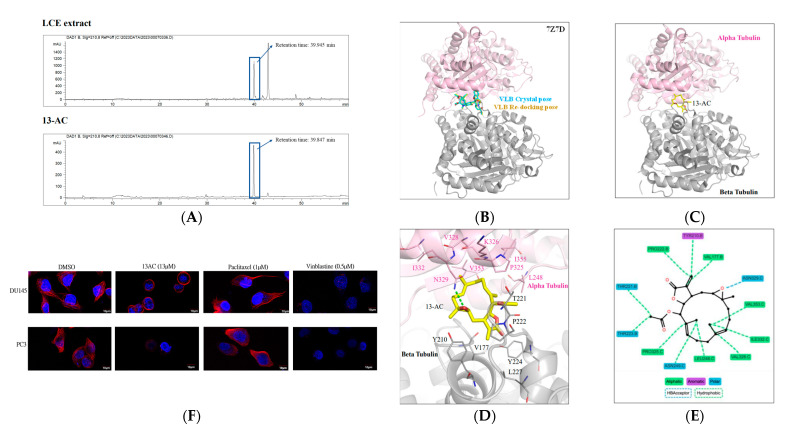
The effect of 13-AC on tubulin depolymerization as demonstrated by means of immunofluorescent and molecular docking assays. (**A**) The HPLC chromatogram of LCE (upper) and 13-AC (lower). (**B**–**E**) Identification of 13-AC on the interaction of αβtubulins with 2D/3D molecular ducking assay. (**F**) The effect of paclitaxel, vinblastine, and 13-AC on tubulin network according to immunofluorescent staining with confocal examination.

Compared with the 1H NMR spectra of 13-acetoxysarcocrossolide (13-AC), the NMR spectra of LCE showed the characteristic signals of 13-AC, including a tertiary methyl group [*δ*_H_ 2.0, (3H-13OAc, s)], olefinic protons at C-17 [*δ*_H_ 5.60 (1H, d, *J* = 2 Hz) and 6.24 (1H, 99 d, *J* = 2 Hz)], and methine proton at C-14 [*δ*_H_ 4.58, (1H, t, *J* = 2.4 Hz)] in α-methyl-γ-lactone ring [34]. Additionally, the HPLC chromatogram of LCE showed a major peak at 39.847 min, coinciding with the retention time of 13-AC (Figure 8A,B). These findings suggested that 13-AC was the major component in the LCE, as demonstrated by the bioactivity-guided fraction.

LCE treatment interfered with the tubulin polymerization and disrupted the cytoskeletal network (Figure 4 and Figure 6F and Table 3). To determine protein–ligand interactions that may indicate the potency of, a docking analysis was performed. Vinblastine served as the co-crystal ligand and was redocked to validate the docking procedure used in the study. The docking pose of vinblastine was similar to the crystal pose, suggesting that a reliable docking procedure was used (Figure 8B). The docking result showed that 13-AC can occupy the binding site, which is sandwiched by the alpha and beta tubulins (Figure 8C). Several interactions with the alpha-tubulin were observed (Figure 8D). A hydrogen bond was formed between 13-AC and the side chain of N329 (Figure 8E). As a ring structure, 13-AC facilitated the hydrophobic interactions with residues L248, N249, P325, V328, I332, and V353 located on the alpha-tubulin. The beta tubulin residues V177, Y210, T221, P222, and T223 supplied the additional hydrophobic interactions (Figure 8E). Together, these interactions sandwiched 13-AC within the tubulin-binding site. The identified interactions would be favorable for 13-AC occupation and could potentially result in the inhibition of tubulin polymerization. Moreover, we further examined the integrity of the cytoskeletal microtubules in PC3 and Du145 treated with 13-AC (10 μg/mL; 13 μM) for 6 h with an immunofluorescent assay. Paclitaxel and vinblastine were the positive polymerization and depolymerization agents of tubulin, respectively. The Du145 and PC3 cells of the control group showed the normal structure of microtubulins. Du145 and PC3 treated with paclitaxel possessed a cluster-like phenomenon of microtubulin; conversely, the cells with 13-AC or vinblastine treatment showed a clear outer edge around the cell nucleus, or the microtubulins disappeared (Figure 8F). The results of the molecular docking assay indicated that 13-AC binds to the same site as vinblastine at 7Z7D of αβ-tubulins, suggesting its potential anti-microtubulin effects by disrupting the equilibrium of the tubulin–microtubulin system and ultimately leading to microtubule depolymerization. SwissADME is a web tool designed for the evaluation of the drug-likeness and pharmacokinetic and medicinal chemistry-related properties of small molecules. It is particularly useful in the early stages of drug discovery to assess whether a compound has the necessary characteristics to become a viable drug candidate. SwissADME provides various predictions and analyses, including physicochemical properties, drug-likeness, pharmacokinetics, and medicinal chemistry parameters [35]. As shown in Table 5, 13-AC demonstrated greater potential in drug development, as it could form favorable chemical bonds with the characteristic of small molecules compared with vinblastine. Gastrointestinal absorption (GIA) and water solubility analysis indicated that 13-AC will be highly absorbed in the gastrointestinal tract and more water-soluble than vinblastine. Combined with its high bioavailability score of 13-AC (0.55), the potential of developing 13-AC for oral administration was suggested, indicating that a larger portion of the administered dose reaches the systemic circulation intact [36]. Additionally, 13-AC is not a substrate for the five major isoforms of cytochrome P (CYP) enzymes. This suggests that it may have reduced toxicity and fewer adverse drug reactions compared to vinblastine, which could be metabolized by these enzymes [37,38]. Moreover, the synthetic accessibility (SA) of 13-AC (5.66) is lower than vinblastine (9.65), and it does not trigger any PAINS 0#Alert, suggesting a solution to synthetic challenges regarding insufficient coral sources and making it suitable for lead optimization. Based on these points, it is suggested that 3-AC holds more potential for development as an oral anticancer drug compared with vinblastine due to its favorable pharmacokinetic and chemical properties.

### 2.9. Effect of LCE on Tumor Growth in Xenograft Human PC3 and Du145 Model

**Figure 9:**
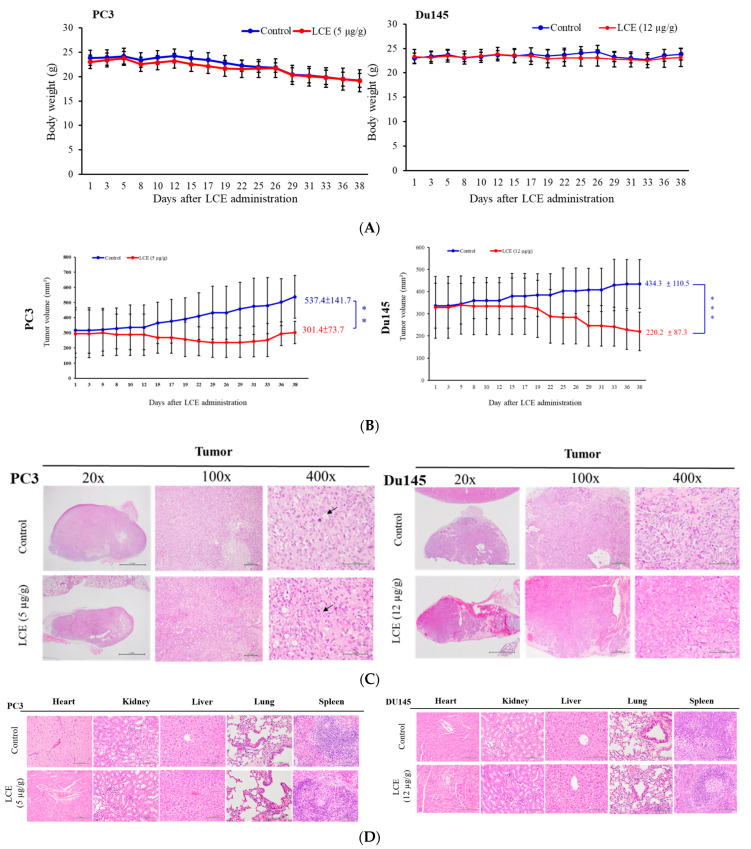
The impact of LCE on tumor growth of PC3 and DU145 in a xenograft model. Tumor-bearing nude mice were fed with solvent control (DMSO), LCE (5 μg/g), and (12 μg/g) for 38 days. (**A**) The body weight and (**B**) tumor volumes were measured every two to three days. Data are shown as mean ± SD. (**C**) Histopathologic features of mice xenograft tumors. Tumor cells expressed round-to-spindly epithelial cells and a high nucleic/cytoplasm ratio with high mitosis (arrow) in both groups. (**D**) No significant lesions of the heart, kidneys, liver, lungs, and spleen were found in either group using H&E staining analysis (400×). (** *p* < 0.01; *** *p* < 0.005).

The above results showed that LCE had a cytotoxic effect on prostate cancer cells (Figure 5A–C), so the impact of LCE on the growth of PC3 and Du145 tumors in xenograft animals was further investigated. PC3 and Du145 cells were injected subcutaneously into the upper right thigh of immunodeficient nude mice until the tumor volume was about 100 mm^3^, and the DMSO or LCE extract was injected intraperitoneally (the dose was PC3: 5 μg/g, Du145: 12 μg/g), and the drug was administered three times a week for 38 days. Comparing the differences between the control group and the LCE extract treatment group, it was found that there was no significant difference between the two groups (Figure 9A). The tumor volume of PC3 and Du145 was about 537.4 ± 141.7 and 434.3 ± 110.5 mm^3^ in the control groups; however, the volumes were 301.4 ± 73.7 and 220.2 ± 87.3 mm^3^ in the LCE group (Figure 9B). The pathological examination of tumor sections in the control groups showed that the cancer cells appeared as round-to-spindly epithelial cells and had a high nucleic/cytoplasm ratio with high mitosis (arrow) in both the control groups of PC3 and Du145 xenograft tumors (Figure 9C). The results of the microscopic examination showed that there were no obvious pathological changes in the heart, kidney, liver, lungs, spleen, and other tissues and organs (Figure 9D).

## 3. Discussion

Marine natural products hold the potential for drug development, but the supply of marine organisms is a major obstacle to the development of marine natural drugs. Although the problem of drug origin can be solved through chemical synthesis technology, the structure of marine natural substances is complex and diverse, and the use of chemically synthesized compounds does not expand chemical diversity. Aquaculture could provide a good growth environment for soft corals, produce active ingredients, and reduce our dependence on marine environments. We have successfully cultivated a large number of corals using artificial breeding systems to establish a drug-derived coral species database, and in 2023, over 180 species of drug-source corals had been collected, which can be mainly divided into six genera, including *Sinularia*, *Lobophytum*, *Sarcophyton*, *Briareidae*, *Capnella*, and *Xenia*. We screened over 180 extracts of artificial aquaculture soft corals and found that 6 extracts exhibited potent cytotoxicity against leukemia cells at a dose of 10 μg/mL (Table 1). In this study, we demonstrated that these corals possessed anticancer active substances by targeting HDAC, topoisomerase II, and tubulin polymerization within the cell-free system (Figure 2, Figure 3 and Figure 4). Therefore, exploring effective therapeutic agents from marine natural products has significant potential.

Metastasis is the leading cause of cancer-related mortality [39], and when cancer cells metastasize to other organs, they need to destroy and cross the basal membrane to metastasize to the adjacent tissues and organs. They also need to invade the extracellular matrix and penetrate the blood vessels to flow with the blood. Our study was performed on more malignant androgen-independent cells, PC3 and Du145. The wound healing and transwell tests confirmed that LCE extract had a very significant inhibitory effect on cell migration and invasion (Figure 6A,B). EMT, on the other hand, is a cellular program in which epithelial cells develop the motor and invasion characteristics that are typical of mesenchymal cells, which is an important initiation of cancer progression, invasion, and metastasis [40]. Therefore, EMT is more important in the process of prostate cancer development. During the EMT process, a series of intracellular proteins are altered, including a decrease in the expression of E-cadherin and an increase in the expression of N-cadherin. Unlike E-cadherin, N-cadherin binds loosely to each other, resulting in cell movement t [40]. On the other hand, during EMT, the cytoskeleton and protein complexes change, which affects cell polarity. In this process, the composition of the intermediate filaments is altered by decreasing cytokeratin and increasing vimentin, which in turn leads to cell motility [41,42]. However, the main initiation signal of EMT is certainly represented by the downregulation of E-cadherin, the expression that decreases during EMT, while the loss of function of this protein promotes this transition. Transcriptional repression of E-cadherin has been considered a critical step during EMT [43]. Combined with the significant anti-metastasis and -invasion effects, LCE may have good potential for anti-prostate cancer cell metastasis.

Transforming growth factor β (TGF-β) is a secreted cytokine that is well known for its role as an EMT inducer [44]. In addition, TGF-β promotes tumor cell motility, survival, invasion, and metastasis and the evasion of immune p53 signaling in advanced cancer cells [44]. Therefore, TGF-β was used as an inducer of EMT to promote the expression of excess EMT in this study. The results of the transwell assay showed that the migration transfer ability of PC3 and Du145 was induced under the action of TGF-β, and the migration transfer of both cell lines was significantly inhibited by LCE after treatment with this extract (2.5 μg/mL) and TGF-β at the same time. The inhibition effect of LCE was not reduced by the addition of TGF-β, indicating that LCE still inhibited the metastasis induced by TGF-β in prostate cancer cells with the enhancement of E-cadherin (Figure 6C,D). A possible factor in previous studies suggested a differential effect of TGF-β on Du145 and PC3 cell activation might be related to phosphatase and tensin homologs (PTEN) in the PI3-kinase/AKT pathway, which inhibits PI3-kinase-dependent AKT phosphorylation. In contrast, PTEN has been shown to mutate in many cancers, which can lead to increased levels of pAKT ^ser473^ expression and increased cell survival [45]. In addition, PTEN was active in Du145 cells but inactive in PC3 cells [46,47], and the protein results also showed that PC3 did not express PTEN protein, while Du145 showed the obvious expression of PTEN protein and increased its expression under LCE (Figure 6E), so it was speculated that TGF-β-induced AKT phosphorylation could not be inhibited by PTEN in PC3 cells. TGF-β showed a more obvious effect on the induction of PC3 cells [48].

Based on the results of the 2D protein analysis, six target proteins which LCE possibly affects were screened (Table 3). Keratin 7 and galactoside alpha-(1,2)-fucosyltransferase 1 were increased in PC3 and Du145 treated with LCE (10 μg/mL), and the protein expression of the two cell lines was also consistently increased following treatment with LCE (Figure 6F). Among them, keratin 7 is a type II keratin in cells, a type II intermediate filament (IF) that forms the cytoskeleton in the cytoplasm, which is present in all mammalians epithelial cells and is specifically expressed in epithelial cells in the lumen of internal organs, as well as in glandular ducts and blood vessels. Keratin is an important protein that protects the structural integrity of the epithelial under stress and is thought to be a regulator of many cellular properties and functions, including apical–basal polarization, motility, cell size, protein synthesis, and membrane trafficking and signaling. In cancer cells, keratin is widely used as a diagnostic tumor marker, because epithelial malignancies largely maintain specific keratin patterns associated with the cells of origin, such as lung, adrenocortical, prostate, and liver cancer, which are negative for keratin 7 and keratin 20 [49]. In addition, previous studies indicated that active keratin is involved in cancer cell invasion and metastasis and treatment [50], so the altered keratin expression caused by LCE may in turn affect the physiology of prostate cancer cells, especially in terms of cancer cell invasion and metastasis. Another increased protein is galactoside alpha-(1,2)-fucosyltransferase 1, which is a protease that catalyzes chemical reactions in physiology and participates in cell metabolism-related reactions, and it has been pointed out that the increase in galactoside alpha-(1,2)-fucosyltransferase 1 can inhibit the metastasis of pancreatic cancer cells by regulating glycoproteins [51]. Dynactin subunit 2 is a multi-subunit protein complex that is essential for the movement of cytoplasmic dynein. It binds to microtubules and cytoplasmic dynein in cells and is involved in a variety of cellular functions, including the transport from the ER to the Golgi apparatus, centripetal movement of lysosomes and endosomes, spindle formation, chromosome movement, nuclear localization, and axonogenesis [52]. Thus, it is interesting to mention that LCE exhibited a potent efficiency in terms of EMT and inhibition invasion of prostate cancer cells, with disruption of the cytoskeleton.

With NGS analysis, the result of the volcano plots showed that the important regulators, HO1 and CLCX1, had a consistent effect in PC3 and Du145 cells with LCE treatment (Figure 7A). CXCL1, also known as GROα (growth-regulated oncogene alpha), is a chemokine that plays a role in inflammation and immune responses. In the context of cancer, CXCL1 has been implicated in various aspects of tumor progression and metastasis, making it a potential target for anticancer therapies [53]. CXCL1 can promote tumor growth by stimulating the proliferation of cancer cells. Additionally, it plays a role in angiogenesis, the process by which new blood vessels are formed to supply nutrients and oxygen to tumors. Inhibiting CXCL1 signaling can therefore impede tumor growth and reduce the tumor’s ability to access essential nutrients [54]. However, this chemokine is involved in the recruitment of immune cells to the tumor microenvironment, where it can promote tumor cell migration and invasion. By targeting CXCL1 or its receptors, it may be possible to inhibit these processes, thereby preventing cancer cells from spreading to other parts of the body [55]. Given its involvement in multiple aspects of tumor progression and metastasis, CXCL1 has emerged as a potential therapeutic target in cancer treatment. Strategies aimed at inhibiting CXCL1 signaling include the use of monoclonal antibodies, small-molecule inhibitors, and gene therapy approaches. These therapies are being investigated in preclinical studies and clinical trials to assess their safety and efficacy in cancer patients [56]. In this study, the overexpression of CXCL1 reduced viability with 16% in LCE-treated Du145 cells (Figure 7B). In summary, CXCL1 plays a multifaceted role in cancer progression and metastasis, making it an attractive target for anticancer therapies. By inhibiting CXCL1 signaling, it may be possible to impede tumor growth, metastasis, and immune suppression, ultimately improving patient outcomes in various types of cancer. The regulation of heme oxygenase-1 (HO-1), also called heat shock protein 32, plays a significant role in cancer therapy due to its role in cellular responses to oxidative stress and inflammation, especially for detoxification and protection [57]. In addition, the enhancement of HO-1 expression suppressed the growth in androgen-dependent LNCap and androgen-independent PC3 and Du145 cells [58]. The knockdown of HO-1 reversed viability with 7.3% in LCE-treated Du145 cells (Figure 7C). Several studies indicated that HO-1 could be a notable therapeutic target in PCa [59,60]. Both HO-1 and CXCL1 play complex and interconnected roles in cancer progression and represent promising targets for anticancer therapies. Understanding the interplay between these factors and their contributions to the tumor microenvironment is crucial for the development of effective treatment strategies that can disrupt tumor growth, metastasis, and immune evasion in cancer.

Tubulin polymerization, the process by which tubulin proteins assemble into microtubules, plays a crucial role not only in cell division but also in cell migration and invasion, particularly in the context of cancer metastasis. The disruption of tubulin polymerization can impair cell polarization and inhibit efficient migration and invasion [61]. In addition, tubulin polymerization can influence various signaling pathways that regulate cell migration and invasion. For instance, microtubule dynamics can affect the activity and localization of signaling molecules such as Rho GTPases, which play key roles in cytoskeletal rearrangements and cell motility. The disruption of tubulin polymerization can alter the activation of these signaling pathways, impacting cancer cell migration and invasion. Targeting tubulin polymerization and microtubule dynamics has therapeutic implications for cancer metastasis [62]. Microtubule-targeting agents (MTAs), such as taxanes and vinca alkaloids, disrupt tubulin polymerization and inhibit cancer cell proliferation [63,64]. The result of our bioactivity-guided fractionation substantiated that 13-AC was the active component in LCE by means of NMR and HPLC identification (Figure 8A) [34], and we targeted alpha and beta tubulins to disrupt the tubulin dynamics using molecular ducking analysis and confocal examination (Figure 8B–E). Specifically, a hydrogen bond is formed between 13-AC and the side chain of the amino acid N329. Hydrogen bonds are important in molecular recognition and the stabilization of protein–ligand complexes. The ring structure of 13-AC allows it to engage in hydrophobic interactions with specific residues on the alpha-tubulin subunit. These residues (L248, N249, P325, V328, I332, and V353) likely have hydrophobic side chains that can interact favorably with the hydrophobic regions of the ligand. In addition to interacting with alpha-tubulin, 13-AC also engages in hydrophobic interactions with residues on the beta-tubulin subunit (V177, Y210, T221, P222, and T223). These interactions contribute to the overall stability of the protein–ligand complex. Overall, this interaction between 13-AC and tubulin subunits suggests a potential mechanism for the binding of 13-AC to tubulin, which could have implications for drug design or understanding the biological effects of 13-AC. Finally, SwissADME is a web tool developed by the Swiss Institute of Bioinformatics for the prediction of the pharmacokinetic properties and drug-likeness of small molecules [35]. When examining 13-AC (13-acetoxysarcocrassolide) using SwissADME, the tool provides insights into its pharmacokinetic properties and drug-likeness. By comprehensively analyzing these factors, researchers can assess the overall potential of 13-AC as an oral anticancer drug compared to vinblastine. This evaluation provides insights into its suitability for further development and optimization in drug discovery programs.

## 4. Materials and Methods

### 4.1. Cell Lines, Chemicals, and Biological Materials

The cell lines, leukemia cells (K562 and Molt4), prostate cancer cells (Du145 and PC3), and normal fibroblasts (CCD-966SK) were purchased from the American Type Culture Collection (ATCC, Manassas, VA, USA). Cells were incubated in a 5% Co2 humidified atmosphere at 37 °C. RPMI 1640 was the growing medium for Molt 4. Minimum essential medium was the growing medium for CCD-966SK, K 562, and Du145. Ham’s F-12 medium was the growing medium for PC3. Glutamine (2 mM), antibiotics (100 µg/mL of streptomycin and 100 units/mL of penicillin), and 10% fetal calf serum (FCS) were used to supplement the growing medium. RPMI 1640, minimum essential medium, Ham’s F-12 medium, glutamine, antibiotics (streptomycin and penicillin), and FCS were obtained from GibcoBRL (Gaithersburg, MD, USA). 3-(4,5-Dimethylthiazol-2-yl)-2,5-Diphenyltetrazolium Bromide (MTT), dimethyl sulfoxide (DMSO), bovine serum albumin (BSA), and all other chemicals were purchased from Sigma-Aldrich (St. Louis, MO, USA). Antibodies against Slug, snail, p-PTEN, XIAP, and HO-1 were purchased from Santa Cruz Biotechnology (Santa Cruz, CA, USA). Anti E-Cadherin and N-Cadherin antibodies were obtained from ABclonal Technology (Taipei, Taiwan). Antibodies against cleaved caspases-3, cytokeraton 1 and 7, HRF, dynactin 2, PSME, and galactoside 2-α-L fucosynltransferase 1 were obtained from Cell Signaling Technologies (Beverly, MA, USA). HRP-conjugated anti-mouse and anti-rabbit antibodies were harvested from Molecular Probes (Eugene, OR). 4′,6-diamidino-2-phenylindole (DAPI) and fluorescence-conjugate anti-rabbit antibody were bought from Invitrogen technologies (Carlsbad, CA, USA). The topoisomerase II drug screening kit, HDAC activity assay kit (Fluorometric), and tubulin polymerization assay kit were purchased fromTopoGen, Inc (Port Orange, FL, USA), Abcam (Cambridge, UK) and Cytoskeleton (Denver, CO, USA), respectively. Cell Tubulin Staining Kit was obtained from AAT Bioquest (Pleasanton, CA, USA).

### 4.2. Preparation of Coral Extracts

The soft coral was first freeze-dried and then finely ground into powder. The powder was subsequently extracted with ethyl acetate using a weight-to-volume ratio of 1:10 at room temperature for 2 h. This extraction process was repeated three times. The resulting extracts were combined and concentrated under reduced pressure to yield the ethyl acetate extract.

### 4.3. In Vitro Proliferation Assay

Cancer cells were seeded into individual wells of a 96-well plate at a density of 7 × 10^4^ cells/well for Du145 and PC3 and 2 × 10^5^ cells/well for CCD-966SK, Du145, and PC3 in various media conditions. Following treatment with extracts, the cells were incubated for 24, 48, or 72 h. After the respective incubation periods, 50 µL of MTT solution was added to each well for staining. The plates were further incubated at 37 °C for 2–4 h. Absorbance readings at 570 nm and 620 nm were obtained using an ELISA reader (Anthoslabtec instrument, Salzburg, Austria). The IC_50_ value, representing the concentration causing 50% inhibition of cell viability, was subsequently calculated based on these absorbance measurements.

### 4.4. Western Blotting Assay

Equal amounts of protein were separated by means of electrophoresis on 10%, 12%, or 15% SDS-PAGE gels and then electrotransferred to PVDF membranes. The membranes were blocked for 30 min with 5% blocking serum in TBS-T buffer. Specific primary antibodies were then applied, followed by specific secondary antibodies to detect protein expression using an enhanced chemiluminescence kit (Pierce, Rockford, IL, USA).

### 4.5. Network Pharmacology with RNASeq Analysis

The total RNA was purified with TruSeq Stranded mRNA Library Prep kit (Illumina, San Diego, CA, USA). A series of sequencings of FastP [65], SortMeRNA [66], HISAT2 [67] and featureCounts [68] were conducted, and further, an array of DGE, GSEA, WGCNA, and function enrichment was used to determine differences in gene expression in the biological process, molecular function, and cellular component (GENOMICS, Taipei, Taiwan).

### 4.6. Activities of HDAC, Topoisomerase II, and Tubulin Polymerization in Cell-Free System

#### 4.6.1. Assay of HDAC Activity in Cell-Free System

According to the manufacturer’s protocol (BPS Biosciences, San Diego, CA, USA), the extracts (including the carboxylic and hydroxamic acids) were screened for their pan-HDAC-inhibitory activity using an HDAC inhibitor drug screening kit. Different concentrations of the extracts were applied, and the samples were incubated for 30 min at 37 °C to assess the deacetylation of the substrate, which sensitized it. The reaction was halted by means of treatment with 10 μL of lysine developer, and the mixture was further incubated for 30 min at 37 °C to generate a chromophore. Fluorescence measurements were then taken using a spectrofluorometer (Biotek synergy, Winooski, VT, USA) at excitation = 350–380 nm and emission = 440–460 nm.

#### 4.6.2. Assay of Topoisomerase II Activity in Cell-Free System

The assay was performed following the manufacturer’s protocol [14,16] and a standard relaxation reaction mix (20 μL) was utilized. This mix contained 50 mM Tris-HCl (pH 8.0), 10 mM MgCl, 200 mM potassium glutamate, 10 mM dithiothreitol, 50 μg/mL bovine serum albumin, 1 mM ATP, 0.3 μg of pHOT1 plasmid DNA, 8 units of human topo II (Topogen, Columbus, OH, USA), and either etoposide (10 mM) or various concentrations of coral extracts. The reaction was carried out at 37 °C for 30 min. The termination of the reaction was achieved by adding 2 μL of 10% SDS, followed by digestion of the bound protein with 2.5 μL of proteinase K (50 μg/mL) and incubation at 37 °C for 15 min. Subsequently, the DNA product was analyzed by means of electrophoresis on a vertical 2% agarose gel at 2 volts/cm in 0.5× TAE buffer, and images were captured using the Eagle Eye II system (Stratagene, La Jolla, CA, USA).

#### 4.6.3. Assay of Tubulin Polymerization in Cell-Free System

According to the manufacturer’s protocol, the extracts (including the carboxylic and hydroxamic acids) were screened for tubulin polymerization. Tubulin protein was incubated with GTP and fluorescent-labeled tubulin in a reaction buffer containing 80 mM PIPES (pH 6.8), 1 mM EGTA, 2 mM MgCl_2_, and 1 mM GTP. The reaction was initiated by adding tubulin to the buffer and monitored over time using a spectrofluorometer (Biotek synergy, Winooski, VT, USA) at excitation/emission wavelengths of 360/420 nm. Changes in fluorescence intensity indicated polymerization activity, which was analyzed to determine the effects of various extracts on microtubule assembly.

### 4.7. Two-Dimensional Electrophoresis

The cell lysate was precipitated by adding 0.11 g of TCA per 1 mL of acetone (TCA solution) and 20 mM DTT, followed by incubation in a −20 °C freezer for 30 min. The precipitate was then washed twice with TCA solution (supernatant removed by means of centrifugation at 12,000 rpm) and air-dried. Next, the sample was rehydrated with rehydration buffer and incubated at 4 °C overnight. After quantification, 125 μL of the sample was taken for isoelectric focusing (IEF). Following IEF, the strip was rinsed with water and then equilibrated in SDS equilibration buffer (containing 0.5 g DTT) for 15 min. The strip was placed on an SDS-PAGE gel, sealed with 0.5% agarose sealing solution, and subjected to two-dimensional electrophoresis. Finally, the proteins were visualized by means of silver staining.

### 4.8. Plasmid and siRNA Transfection

Empty vector (EV) pCMV6-AC-GFP and human CXCL1- pCMV6-AC-GFP plasmids (Origene; Rockville, MD, USA) were used for transfection. Cells were grown in a 10 cm dish (2 × 10^6^ cells/dish) for 24 h before transfection, and 5 μg of EV or Human CXCL1- pCMV6-AC-GFP plasmids was transfected using a Lipofectamine 3000 transfection reagent (Invitrogen) following the manufacturer’s instructions. After 30 h of transfection, cells were harvested and plated in 96-well culture plates (7000 cells/well) and 10cm dishes (1 × 10^6^ cells). Cells were treated with LCE for viability analysis and Western blotting after 24h. For siRNA transfection, control siRNA and human HO-1 siRNA were purchased from Origene (Rockville, MD, USA). Cells were seeded at a density of 1.5 × 10^5^ cells/mL in a growth medium for 24 h. Cells were transfected with Lipofectamine RNAiMAX reagent following the manufacturer’s protocol. At 48 h after transfection, cells were subcultured and seeded in 96-well culture plates (7000 cells/well) and 10 cm dishes (2 × 10^6^ cells). After 24 h, cells were treated with LCE for viability analysis and Western blotting.

### 4.9. Immunostaining Confocal Examination

The cells were seeded 7 × 10^4^ cells/mL into 12-well culture plates, and each well had a sterile circular coverslip in the growth medium. After 24 h, cells were treated with DMSO, LCE 2.5 µg/mL, LCE 2.5 µg/mL + TGF-α 5 ng/mL, and TGF-α 5 ng/mL, respectively, for 6 h, followed by immunostaining. For immunostaining and confocal imaging, the cells were rinsed with PBS, followed by fixation with 1% paraformaldehyde for 30 min at room temperature on a shaker. Then, the cells were rinsed three times with PBS and incubated in 0.1% Triton X-100 in PBS for 15 min. The cells were incubated in 300 µL of 1% BSA (bovine serum albumin) (Sigma-Aldrich, Melbourne, Australia) in PBS for 30 min, followed by incubation with the primary antibodies (E-cadherin and N-cadherin; 1:100) overnight at 4 °C. At the end of the incubation step, the cells were washed thrice with PBS, and then the cells were incubated for 1 h with the fluorescence-labeled goat anti-rabbit secondary antibody (Invitrogen) at 1:500 and DAPI 1:1000 in 1% BSA. The cells were washed three times with PBS for 5 min each. Finally, the coverslips were mounted with a fluoro-shield mounting medium (Sigma Aldrich) and sealed with nail polish. The fluorescence images were acquired using an Olympus IX-81 motorized inverted microscope with an FV-1000 laser scanning confocal system using the confocal laser scanning microscope LSM-800 (ZEISS, Göttingen, Germany). Cells were seeded 7 × 10^4^ cells/mL into 4 chambers of Glass-Bottomed Dishes (Cellvis; Sunnyvale, CA, USA). After 24h, the cells were treated with DMSO, LCE, Paclitaxel (1 µM), and Vinoblastin (0.5 µM), respectively, for 6 h, followed by immunostaining. After washing with PBS, the cells were stained with tubulin using a Cell Tubulin Staining Kit (AAT Bioquest, Pleasanton, CA, USA) according to the manufacturer’s protocol. The fluorescence images were acquired using an Olympus IX-81 motorized inverted microscope with an FV-1000 laser scanning confocal system using the confocal laser scanning microscope LSM-800 (ZEISS, Germany).

### 4.10. Molecular Docking and Interaction Analysis

The compound, 13-AC, was molecularly docked using the Schrödinger Maestro molecular software v12.1 suite [69]. The protein structure was obtained from the Protein Data Bank (PDB ID: 7Z7D) repository. Protein preparation and compound preparation were performed using the Maestro Protein Preparation and LigPrep modules in Maestro, respectively. The co-crystal ligand, vinblastine (VLB), was used to define the binding site. Docking analysis was performed using Glide [70]. The extra-precision (XP) setting was used. All other constraint settings were set to default. The docking procedure was validated by redocking the co-crystal ligand, vinblastine, and comparing its spatial positioning to the resulting docking pose. Protein–ligand interactions and the 2D interaction diagram were generated using the ProLIF Python package [71]. The final binding poses were rendered using PyMOL v.2.5.4 software.

### 4.11. Antitumor Examination of LCE with Xenograft Human Prostate Cancers

The xenograft animal model was established following the existing literature [16]. Five-week-old nude mice (BALB/cAnN.Cgfoxnlnu/CrlNarl) were purchased from the National Laboratory Animal and Research Center. The Animal Care and Treatment Committee of the National Museum of Marin Biology & Aquarium (IACUC Permit Number 201707) approved this study. The Guide for the Care and Use of Laboratory Animals of the National Institutes of Health was followed in all experiments. All efforts were made to minimize animal stress/distress. Human prostate cancer PC3 and Du145 cells were injected subcutaneously into the right leg of the nude mice. LCE was administered by means of intraperitoneal injection in the experimental group, while the control group received solvent only (DMSO). The drug was administered three times a week. The tumor volume was calculated using the following formula: width^2^ × length/2. After 38 days of treatment, animals were euthanized with carbon dioxide according to standard protocols. Tumors, heart, liver, spleen, lungs, and kidneys were collected post-euthanasia. The tumor weights were measured, and the organs were fixed with formalin for histochemical staining and microscopic examination.

### 4.12. Statistical Analysis

All statistics are expressed as the mean ± standard deviation (SD). The comparison of statistically significant data was performed using Student’s independent t-test at *p* < 0.05, *p* < 0.01, and *p* < 0.001 for each experiment.

## 5. Conclusions

As is widely known, soft corals serve as excellent sources of marine-derived natural products. Among them, genera such as Sarcophyton, Sinularia, and Lobophytum are particularly attractive targets for marine natural product research. Six corals were shown to be capable of potent inhibition of the activities of cell growth, HDAC, topoisomerase II, and tubulin polymerization based on screening more than 180 EA extracts of marine soft corals. Particularly, the coral *Lobophytum crassum* exhibited the most effective enhancement of cytotoxicity and suppression of invasion in PCs. Our findings showed that these EA extracts were enriched with active compounds to develop potent inhibitors of HDAC, topoisomerase II, and tubulin polymerization in a cell-free system. Moreover, our research unveils that 13-AC could be a potential candidate with high gastrointestinal absorption (GIA), water solubility, bioavailability score, and synthetic accessibility for cancer therapy. However, the source of marine natural products is a serious problem and a major obstacle to supplying active compounds for the development of clinical drugs. Many still believe that the total synthesis of marine compounds could not provide a sufficient amount for clinical trials. The successful aquaculture of marine corals is an exciting development and represents a potential advancement in the acceleration of lead drug development. Given that these compounds are recognized for their broad spectrum of biological activities, they hold considerable potential for the development of various pharmaceuticals that are beneficial to human health and ecology.

## Figures and Tables

**Table 1:** Cytotoxic activity of six coral extracts against different leukemia and prostate cancer cell lines after 72 h using MTT assay. The IC_50_ values were calculated using CalcuSyn software Version 1.1.1.

	IC_50_ (μg/ mL), 72 h			
	Leukemia Cell		Prostate Cancer	
Crude Extracts	Molt 4	K562	Du145	LNCaP
**C1**	4.11 ± 0.99	4.58 ± 0.15	12.39 ± 2.11	17.37 ± 3.86
**C2**	<0.16	0.33 ± 0.05	14.66 ± 0.79	15.49 ± 4.56
**C3**	1.87 ± 0.36	6.11 ± 2.28	11.44 ± 4.26	36.18 ± 0.69
**C4**	0.66 ± 0.084	2.65 ± 0.44	36.9 ± 2.8	37.88 ± 0.21
**C5**	1.5 ± 0.7	2.29 ± 0.17	4.96± 0.04	2.76 ± 0.34
**C6**	1.57 ± 0.57	2.27 ± 0.12	5.88 ± 0.48	9.17 ± 0.07

**Table 2:** Cell growth inhibition of LCE against various prostate cancer cell lines after 24 and 72 h using MTT assay. Values of IC_50_ were calculated using calcuSyn software version 1.1.1.

	Prostate Cencer			Fibroblast
	LNcap	PC-3	Du145	CCD966SK
24 h	5.70 ± 1.37	4.14 ± 0.48	5.27 ± 2.19	>20
72 h	2.76 ± 0.34	1.92 ± 0.09	4.96 ± 0.04	8.21 ± 1.14

**Table 3:** Identification of target proteins in PC3 and Du145 treated with LCE by means of proteomic and LC-MS/MS assays.

	Du145		PC3	
Protein	DMSO	LCE	DMSO	LCE
Keratin, type II cytoskeletal 7	0.06	0.30	0.53	1.92
Dynactin subunit 2 isoform 1	1.76	1.37	0.53	0.34
Keratin 1	0.61	0.18	1.78	1.44
Proteasome activator complex subunit 1 isoform 1	1.82	1.14	0.60	0.44
Galactoside α-(1,2)-fucosyltransferase 1	0.10	0.66	1.21	2.03
Translationally-controlled 1	0.62	0.49	1.85	1.04

**Table 4:** Top 10 pharmacological targets of PC3 and Du145 with DMSO and LCE (10 μg/mL) treatment, respectively, using NGS analysis.

PC3 (LCE/DMSO)			Du145 (LCE/DMSO)		
Symbol	Log^2^ Fold Change	padj	Symbol	Log^2^ Fold Change	padj
HMOX1	5.56	0	HMOX1	7.46	0
CXCL1	−5.35	0	HSPA1A	5.58	0
DDIT3	5.46	0	HSPA1B	5.50	0
HSPA1A	5.10	0	HSPA6	6.53	0
ATF3	5.41	0	CXCL1	−4.30	0
ULBP1	5.52	0	DUSP1	4.04	0
HSPA1B	4.34	0	BAG3	3.79	0
OSGIN1	5.34	0	DNAJB4	3.76	0
SESN0	4.69	0	DNAJB1	3.54	0
CHAC1	5.7	0	TXNIP	−3.79	0

Heme oxygenase 1 (HMOX1); C-X-C motif chemokine ligand 1 (CXCL1); DNA damage inducible transcript 3 (DDIT3); heat shock protein family A (Hsp70) member 1A (HSPA1); activating transcription factor 3 (ATF3); UL16 binding protein 1 (ULBP1); heat shock protein family A (Hsp70) member 1B (HSPA1B); oxidative stress-induced growth inhibitor 1 (OSGIN1); Sestrin 2 (SESN0); ChaC glutathione-specific gamma-glutamylcyclotransferase 1 (CHAC1); heat shock protein family A (Hsp70) member 6 (HSPA6); dual-specificity phosphatase 1 (DUSP1); BAG cochaperone 3 (BAG3); DnaJ heat shock protein family (Hsp40) member B4 (DNAJB4); DnaJ heat shock protein family (Hsp40) member B1 (DNAJB1); thioredoxin interacting protein (TXNIP).

**Table 5:** Pharmacokinetic and drug-likeness predictions of vinblastine and 13-AC with SwissADME.

	MW	Water Solubility	GIA	BBB	Log K_p_	Bioavailability Score	PAINS#alerts	Synthetic Accessibility
Vinblastine	810.97	Poorly	Low	No	−8.49	0.17	0	9.65
13-AC	374.47	Soluble	High	Yes	−6.39	0.55	0	5.66

## Data Availability

The data presented in this study are available on request from the corresponding author.
